# Renal Protective Effects of *Inonotus obliquus* on High-Fat Diet/Streptozotocin-Induced Diabetic Kidney Disease Rats: Biochemical, Color Doppler Ultrasound and Histopathological Evidence

**DOI:** 10.3389/fphar.2021.743931

**Published:** 2022-01-17

**Authors:** Yan Zhang, Hui Liao, Dayue Shen, Xilan Zhang, Jufang Wang, Xiaohong Zhang, Xiaocheng Wang, Rongshan Li

**Affiliations:** ^1^ Department of Nephrology, Fifth Hospital of Shanxi Medical University (Shanxi Provincial People’s Hospital), Taiyuan, China; ^2^ Department of Pharmacy, Fifth Hospital of Shanxi Medical University (Shanxi Provincial People’s Hospital), Taiyuan, China; ^3^ School of Pharmacy, Shanxi Medical University, Taiyuan, China; ^4^ Department of Ultrasonic Diagnosis, Fifth Hospital of Shanxi Medical University (Shanxi Provincial People’s Hospital), Taiyuan, China; ^5^ Department of Statistic and Medical Record, Fifth Hospital of Shanxi Medical University (Shanxi Provincial People’s Hospital), Taiyuan, China

**Keywords:** *Inonotus obliquus*, diabetic kidney disease, biochemical analysis, color Doppler ultrasound, histopathology

## Abstract

Diabetic kidney disease (DKD) is the current leading cause of end-stage renal disease. *Inonotus obliquus* (chaga), a medicinal fungus, has been used in treatment of diabetes. Here, we aim to identify the renal protective effects of chaga extracts on a DKD rat model which was induced by a high-fat diet and streptozotocin injection. During the total 17-weeks experiment, the biological parameters of serum and urine were examined, and the color Doppler ultrasound of renal artery, the periodic acid-Schiff staining, and electron microscopy of kidney tissue were performed. The compositions of chaga extracts were analyzed and the intervention effects of the extracts were also observed. Compared with the normal control group, the biochemical research showed that insulin resistance was developed, blood glucose and total cholesterol were elevated, urinary protein excretion and serum creatinine levels were significantly increased in the DKD model. The ultrasound examinations confirmed the deteriorated blood flow parameters of the left renal interlobar artery in the rat models. Finally, histopathological data supported renal injury on the thickened glomerular basement membrane and fusion of the foot processes. 8 weeks intervention of chaga improved the above changes significantly, and the 100 mg/kg/d chaga group experienced significant effects compared with the 50 mg/kg/d in some parameters. Our findings suggested that Doppler ultrasound examinations guided with biochemical indicators played important roles in evaluating the renal injury as an effective, noninvasive, and repeatable method in rats. Based on biochemical, ultrasound, and histopathological evidence, we confirmed that chaga had pharmacodynamic effects on diabetes-induced kidney injury and the aforementioned effects may be related to delaying the progression of DKD.

## Introduction

Diabetic kidney disease (DKD), one of the most severe complications of diabetes mellitus (DM), is currently a leading cause of the end-stage renal disease (ESRD) (Brosius et al., 2009; Azushima et al., 2018; Giralt-Lopez et al., 2020). It is reported that more than 40% of patients with DM will eventually develop DKD (KDIGO Executive Committee, 2020). Accordingly, there is an urgent need to prevent or treat kidney failure in diabetic patients. Some previous studies have shown that nontoxic biological macromolecules, mainly polysaccharides from natural sources, possess prominent efficacies on DM (Wang et al., 2016). The polysaccharides from *Inonotus obliquus* (chaga), a white-rot fungus belonging to the family Hymenochaetaceae, were reported to have antidiabetic activities and ameliorate glucolipotoxicity-induced renal fibrosis in diabetic mice (Chou et al., 2016; Wang et al., 2017a). In this article, we showed further interest in finding more evidence about chaga on renal protection in DKD rats.

The availability of animal models is essential for pathological and preclinical research on DKD therapies (Azushima et al., 2018). In 2009, the Animal Models of Diabetic Complications Consortium (AMDCC) defined the criteria for the validation of DKD murine models (Brosius et al., 2009). The main measurement indicators include a decline in glomerular filtration rate (GFR), an increase in albuminuria, the thickening of the glomerular basement membrane (GBM) and the presence of advanced mesangial matrix expansion, arteriolar hyalinosis, and tubulointerstitial fibrosis (Brosius et al., 2009). A noninvasive, robust, and reproducible method to image arteriolar microangiopathy is provided with renal Doppler ultrasound (RDUS), which can give better insight into the structure, function, and physiology of an animal’s kidney (Meyer et al., 2021).

In clinical practice, RDUS provides an accurate renal indication as a significant auxiliary diagnostic technique (Li et al., 2020) and more deterioration predictors on renal functions in DKD patients (Di Nicolo and Granata, 2019). With RDUS, it will be particularly helpful to elucidate whether renal function is aggravated in any of these DKD models. In the current study, we established a DKD rat model guided by biochemical indicators, RDUS assessment, and verified by histopathological evidence. Based on our previous study (Liao et al., 2020), we observed the effects of chaga extracts on the above model.

## Methods

### Preparation of Chaga Extracts

**FIGURE 1:**
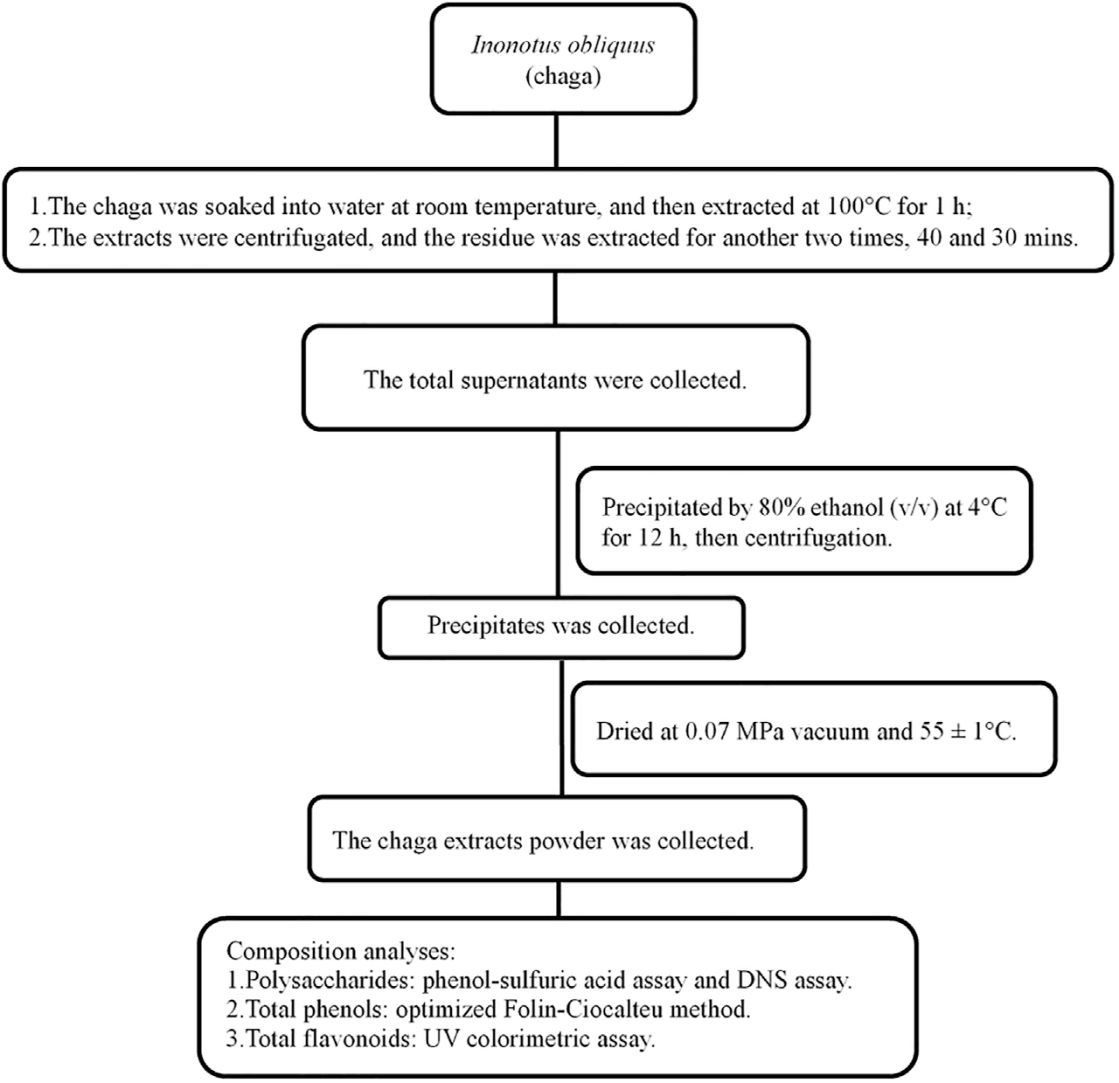
Flowchart of *Inonotus obliquus* extracts and composition analyses. DNS, dinitrosalicylic acid.

Chaga was collected from Huzhong National Nature Reserve of the Greater Khingan range in Heilongjiang province. The process for extracting chaga was shown in Figure 1 and described in brief as follows: 2–3 cm^2^ chaga was soaked in water at 1:10 (w/w) at room temperature for 3 h, and then boiled at 100°C for 1 h. The supernatant was removed and the residue was extracted two other times. The total supernatant was collected and precipitated by 80% ethanol (v/v) at 4°C for 12 h and finally the powder was obtained. Its composition was first determined before it was tested in DKD rat models (Liao et al., 2020).

### Composition Analyses of Chaga Extracts

The total polysaccharide contents (TPCCs), total polyphenol contents (TPCs) and total flavonoid contents (TFCs) in the above extracts were analyzed according to current literatures (Song et al., 2016; Liao et al., 2020; Zhao et al., 2020; Fanali et al., 2021), and described in brief as follows.

#### Analyses of TPCCs

TPCCs were the reducing sugar subtracted from the total sugar content. Total sugar was tested with a phenol-sulfuric acid method using glucose as the standard (Liao et al., 2020), and reducing sugar was determined by 3,5-dinitrosalicylic acid (DNS) assay (Song et al., 2016).

##### Preparation of Total Sugar Standard Curve

1.0 mg/ml D-glucose (Solarbio, Beijing) stock solution was pipetted in deionized water at final concentrations of 25, 30, 35, 40, 45, 50, 55, and 60 μg/ml in 2 ml total volume and mixed with 2 ml of 5% phenol solution (v/v) and 10 ml of concentrated sulfuric acid (Shidande, Shanghai, P.R. China) separately. The mixture was placed in a water bath at 80°C and kept for 30 min. It was then cooled to room temperature, and the A values were measured at 486 nm using a spectrophotometer (Persee, Beijing). The standard curve of total sugar was obtained using the A value as the ordinate and the concentration as the abscissa.

##### Preparation of Reducing Sugar Standard Curve

50 mg of glucose (Solarbio, Beijing) was weighed accurately and dissolved in 100 ml deionized water, and 0.5 mg/ml glucose standard solution was prepared. 0.0, 0.6, 0.8, 1.0, 1.2, and 1.4 ml of the prepared glucose standard solution were added to 2.0, 1.4, 1.2, 1.0, 0.8, and 0.6 ml deionized water respectively, and mixed with 1.5 ml DNS (Shidande, Shanghai, P.R. China) reagent separately. The mixtures were boiled in a water bath at 100°C for 5 min, and then quickly cooled with running water, diluted to 10 ml, and the absorbances were measured at a wavelength of 540 nm. A standard curve of reducing sugar was drawn using glucose concentration as the ordinate and absorbance as the abscissa.

##### Determination of Total Sugar and Reducing Sugar

The samples were accurately weighed and dissolved in deionized water, and 0.2 mg/ml solution of the samples was made separately. Then, 2 ml of the 0.2 mg/ml solution was mixed as aforementioned. The values of total sugar and reducing sugar were calculated according to the standard curve obtained above.

#### Analyses of TPCs

TPCs were analyzed using the Folin-Ciocalteu method which was optimized by response surface methodology (Fanali et al., 2021).In brief, 20 µl of 1 mg/ml sample was mixed with 100 µl of Folin–Ciocalteu’s reagent and 1,580 µl of 50% EtOH. The mixture was kept in the dark for 10 min. Then, 300 µl of an aqueous solution of 0.2 g/ml Na_2_CO_3_ was added and put back in the dark for 2 h with continuous stirring. Finally, the mixture was centrifuged at 10,000 g for 3 min and 200 µl of the sample was put in a Greiner microplate (Solarbio, Beijing). The absorbance was measured with the Infinite M200 PRO microplate spectrophotometer (Tecan Trading AG, Switzerland) at 765 nm. The concentration of the samples was calculated according to a calibration curve made using gallic acid as an analytical standard.

#### Analyses of TFCs

TFCs were determined with UV colorimetric assay (Zhao et al., 2020). In brief, 0.5 ml of chaga extracts was added to 1 ml of sodium nitrite, and allowed to sit for 6 min, 1 ml of 10% aluminum nitrate was then added, and allowed to sit for another 6 min. Then, 10 ml of 1.0 M sodium hydroxide was added to the above mixture, the volume was made up to 20 ml by adding water, and the solution was kept for 15 min. Finally, UV-VIS spectrometry (UH4150, Guangzhou, China) was used to detect the absorbance at 510 nm. The standard curve of the absorbance value of rutin (Product number: YZ100080, purity >95%, Beijing Suolaibao Technology Co. LTD., Beijing, China) concentration solution was then determined. TFC was indicated as mg of rutin equivalent per g of weight of the extract after drying.

### Animal Models, Grouping and Treatment

Sprague Dawley rats (male, 5-week-old, 130 ± 20 g) were purchased from the Laboratory Animal Center, Fifth Hospital of Shanxi Medical University (Shanxi Provincial People’s Hospital). All animal experimental procedures were performed in accordance with the guidelines of the Ethics Review Committee for Animal Experimentation of Shanxi Provincial People’s Hospital (Approval No. 2021-004). All efforts were made to minimize the suffering of the animals. After free access to food and water at a controlled temperature (24 ± 2°C) and humidity (50 ± 5%) for 1 week, the rats were randomly assigned to the following four groups (*n* = 8 per group).

DKD model group: SD rats were fed with high-fat chow (35.5% fat, 20.6% protein and 43.9% carbohydrates, Beijing Boaigang Biotechnology Co. LTD., China) for 8 weeks. After insulin resistance was confirmed, the rats were intraperitoneally injected with streptozotocin (STZ, Sigma, United States) in a single dose of 35 mg/kg. Random blood glucose (RBG) was measured after 72 h of STZ injection, and the rats were confirmed to be type 2 DM (T2DM) when their RBG levels were ≥16.7 mmol/L for three consecutive times (Naidoo and Islam, 2014; Huang et al., 2019). The rats were fed with the high-fat diet and 0.25 ml/100 g tap water by gavage once a day for 8 weeks until the end of the study.

**FIGURE 2:**
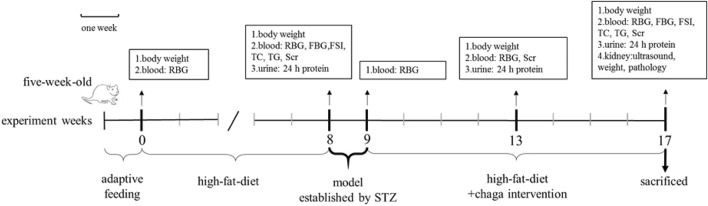
Intervention of chaga extracts on high-fat diet/STZ-induced DKD rats. RBG, random blood glucose; FBG, fasting blood glucose; FSI, fasting serum insulin; TC, total cholesterol; TG, triglyceride; SCr, serum creatinine; STZ, streptozotocin; DKD, diabetic kidney disease.

Chaga groups: The rats in the chaga intervention groups were given chaga extracts obtained from Figure 1 by gavage in a dose of 50 mg/kg (Chaga50) and 100 mg/kg (Chaga100) respectively, once a day for 8 weeks. The interventions started when the T2DM model was established just as the DKD model described above. While using the chaga extracts, the two groups continued to feed on the high-fat diet. Figure 2 showed the procedure of chaga intervention in high-fat diet/STZ-induced DKD rats.

Normal control group: After being fed with ordinary chow (12.0% fat, 20.6% protein and 67.4% carbohydrates, Beijing Boaigang Biotechnology Co. LTD., China) for 8 weeks, the rats were intraperitoneally injected with an equivalent volume of buffer. The rats continued ordinary chow and were given the equal volume of tap water by gavage once a day for another 8 weeks.

### Biological Parameters

#### The Ratio of Kidney Weight/Body Weight

Body weight (BW) was measured at the baseline of (0th week), 8th, 13th, and 17th week. Kidney weight (KW) was measured after the rats were sacrificed in the 17th week. The ratio of KW/BW (Wang et al., 2018) of each rat was calculated and the average ratio was determined per group.

#### Blood Biological Parameters

RBG was detected at the baseline of 8th, 9th, 13th, and 17th week. Serum creatinine (SCr) was detected at the 8th, 13th, and 17th week. Triglyceride (TG), total cholesterol (TC), fasting blood glucose (FBG) and fasting insulin were tested at the 8th and 17th week. The blood glucose level was analyzed using Roche ACCU-CHEK Performa (Roche Diabetes Care GmbH, Mannheim, Germany). The plasma insulin was measured with an insulin ELISA kit (Nanjing Jiancheng Bioengineering Institute, Nanjing, China) and the plasma SCr, TG, and TC were measured by the ISE AU5800 biochemistry analyzers. The insulin resistance index (IRI) at the 8th and 17th week was calculated using the HOMA-IR formula (Wang et al., 2018; Huang et al., 2019):

$$IRI\;=\;\frac{FBG\;\left(mmol/L\right)\times fasting\;insulin\;\left(mIU/L\right)}{22.5}$$

#### Urine Biological Parameters

At the 8th, 13th, and 17th week, 24 h urine was collected, and urinary protein excretion was determined. The rats were placed in metabolic cages (Suzhou Fengshi Laboratory Animal Equipment Co. Ltd., Suzhou, China) without food but with water for 24 h to collect all the urine. All samples were then stored in a −80°C freezer for subsequent analysis. Urine protein level was detected using commercial kits (Nanjing Jiancheng Bioengineering Institute, Nanjing, China).

### Color Doppler Ultrasonography of Renal Arteries

Doppler ultrasonography was performed with MyLab 60 equipment (Esaote, Genova, Italy), equipped with a 4–13 MHz transducer (LA523). All groups underwent ultrasound examination one after the other by the same sonographer at the 17th week before being sacrificed, while another experienced sonographer supervised the examination. Before the ultrasound examination, the animals were fasted for 6 h, and were anesthetized by intraperitoneal injection of pentobarbitone sodium (Beijing Solarbio Technology Co., Ltd., Beijing, China) in a dose of 35 mg/kg.

The lower abdomens were depilated and cleaned, and a proper amount of ultrasonic couplant was used. The probe was gently placed on the lower abdomens of the rats. The position and angle of the probe, and the depth, gain and focus of the ultrasonic image were adjusted accordingly. The color Doppler procedure was performed to display the blood flow of the main renal artery, intersegmental artery, and interlobar artery and all animals were tested on the left kidney. Hemodynamic parameters were measured by using a pulse Doppler with a sampling volume of 0.5 mm and an angle less than 60°. After obtaining a stable spectrum of blood flow, peak systolic velocity (PSV), end-diastolic flow velocity (EDV), mean velocity (MV), systolic acceleration (SAC), systolic acceleration time (SAT) of the above arteries were measured, respectively. The pulsatility index (PI) was calculated according to the following formula (Zou et al., 2017):

$$PI=\frac{PSV-EDV}{MV}$$

and resistive index (RI) also was calculated as follows (Zou et al., 2017; Petrucci et al., 2018):

$$RI=\frac{PSV-EDV}{PSV}$$

All measurements were carried out in triplicate in every rat, and the average was further calculated.

### Histological Analysis

#### Histological Analysis With Light Microscopy

Freshly dissected kidneys were fixed overnight using a 10% neutral formalin buffer, embedded in paraffin, and cut into 3 μm-thick sections with Leica microtome. The sections were stained with periodic acid-Schiff (PAS) reagent and morphological changes were scanned by a KF-PRO-005-EX digital scanner (KFMI, China). Twenty images of glomerular maximal profiles with a vascular pole and/or urinary pole were randomly selected using K-Viewer (1.5.3.1) image analysis software (×400, KFMI, China) and morphological changes were examined and analyzed using Image-Pro plus 6.0 (Media Cybernetics, Maryland, United States).

The length (μm) of the two longest perpendicular diameters in every glomerular capillary tuft without Bowman’s space was measured, and then the mean value was calculated. The areas of the glomerular mesangial region and capillary tuft were also measured, and the relative area of the mesangial region (%) was calculated according to the formula (Wang et al., 2018):

$$Relative\;area\;of\;the\;mesangial\;region\;=\;\frac{area\;of\;the\;mesangial\;region}{area\;of\;the\;capillary\;tuft}\times 100\%$$

#### Histological Analysis With Electron Microscopy

Freshly dissected kidneys were fixed overnight using 2.5% glutaraldehyde and processed according to the standard techniques. The ultrathin sections were stained with uranium acetate-lead citrate for electron microscopy. For each specimen, ten photographs (×20,000 magnification) covering different regions in the glomerular cross section were taken separately.

The thickness of the GBM, the length (μm) of the peripheral GBM, and the number of slit pores were all measured under the electron microscope. Images were selected and analyzed using the RADIUS Control & Imaging software (EMSIS ASIA, Germany). The average of the foot process width ( $\bar{W}FP$ ) was calculated as follows (Wang et al., 2018):

$${\bar{W}}_{P}^{F}\;=\;\frac{\pi }{4}\times \frac{\sum GBM\;length}{\sum slits}\;$$

### Statistical Analysis

GraphPad Prism 9.0 software was used for statistical analysis. All the data of continuous variables were expressed as mean ± standard error of the mean. Two-way analysis of variance (ANOVA), one-way ANOVA and student’s t-test were used to detect the statistical significance. All the reported *p* values were two-tailed, and a *p* < 0.05 was considered statistically significant.

## Results

During the experiment, two rats died in the Chaga50 group, and one died in both the DKD model group and Chaga100 group. Therefore, at the end of the experiment, the data of the remaining twenty-eight rats (eight in the control, seven in the DKD, seven in the Chaga100, and six in the Chaga50 group) were statistically analyzed.

### The Composition of Chaga Extracts

Among three tested ingredients, the polysaccharide content was the highest, which was 18.21 mg/g, total phenols ranked second, 11.32 mg/g, while flavonoid content was the lowest, 2.76 mg/g. The total amount of the three compositions mentioned was 32.29 mg/g.

### Effects of Chaga Extracts on Biological Parameters

#### Influences of Chaga Extracts on BW

**FIGURE 3:**
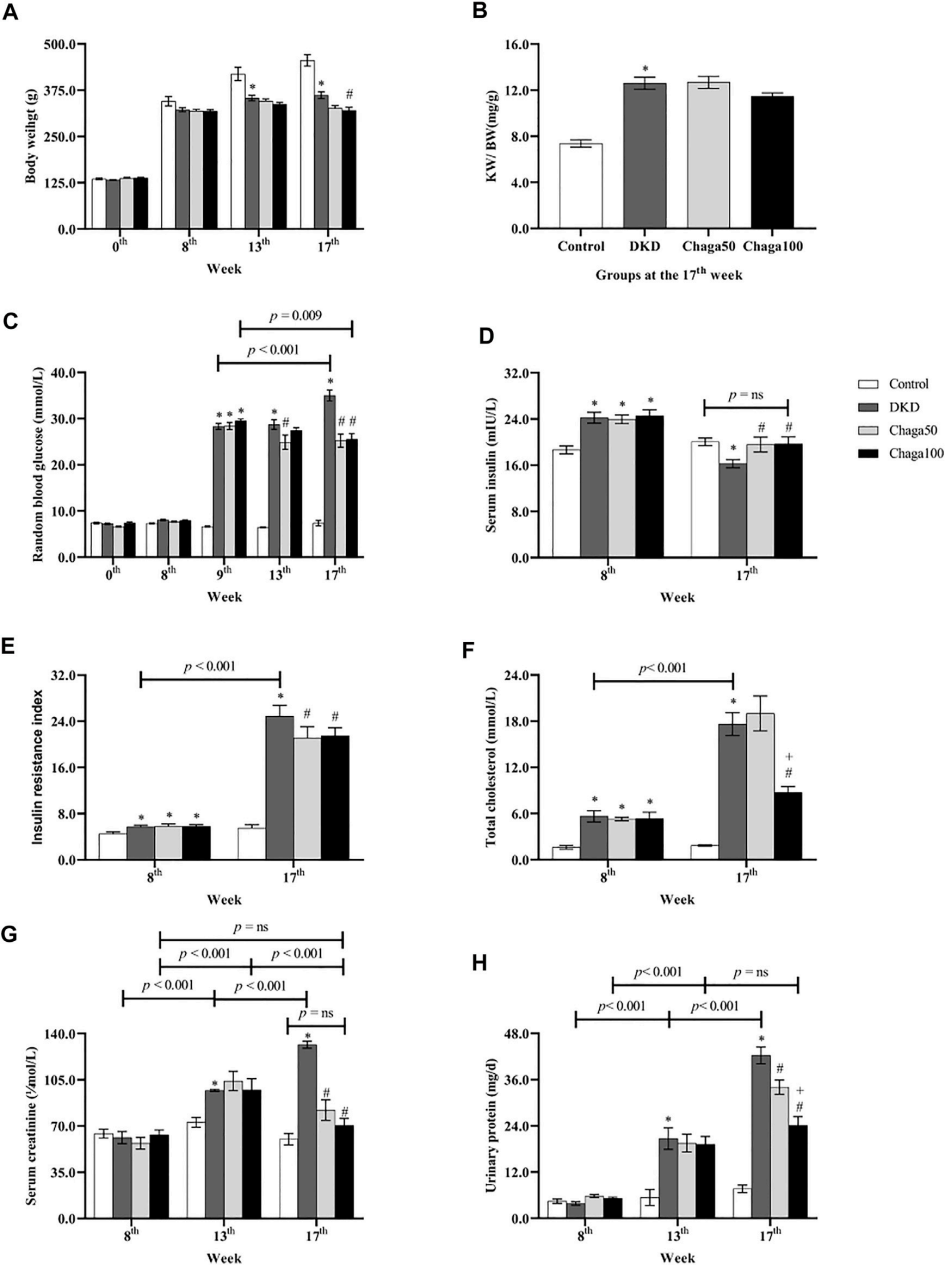
Biological parameters among four groups in different week. **(A)** Body weight of rats in each group. **(B)** The ratio of KW/BW of rats in each group. **(C)** Blood glucose of rats in each group. **(D)** Serum insulin of rats in each group. **(E)** Insulin resistance index of rats in each group. **(F)** Total cholesterol of rats in each group. **(G)** Serum creatinine of rats in each group. **(H)** Urinary protein excretion of 24 h in each group. Data were presented as mean ± SE. DKD, diabetic kidney disease; Chaga50, treatment with 50 mg/kg of chaga group; Chaga100, treatment with 100 mg/kg of chaga group; KW/BW, kidney weight/body weight. *p < 0.05 vs. Normal control group, #*p* < 0.05 vs. DKD group, + *p* < 0.05 vs. Chaga50 group.

As shown in Figure 3A, the baseline BW among the four groups had no significant difference (*p* = ns). After being fed with the high-fat diet for 8 weeks, the BW of the DKD model group and the two chaga-treated groups did not show any difference compared with the normal control group which was fed with ordinary chow (*p* = ns).

At the 13th week, the BW of the DKD model group was significantly lower than that of the control group (*p* < 0.001). There was no significant difference in BWs of the DKD, Chaga50 and Chaga100 groups (*p* = ns).

At the 17th week, the BW of the DKD model group was still significantly lower than that of the control group (*p* < 0.001). The BW of the Chaga100 group (treated with 100 mg/kg of chaga) was significantly decreased compared to the DKD group (*p* = 0.01) (Figure 3A).

#### Influence of Chaga Extracts on the Ratio of KW/BW

The results of KW/BW in the four groups were demonstrated in Figure 3B. At the end of the experiment, the KW/BW was 12.6 ± 0.5 mg/g in the DKD group which was significantly higher than that of the normal rats (7.4 ± 0.3 mg/g) (*p* < 0.001). The KW/BW in the Chaga100 group (11.5 ± 0.3 mg/g) was lower than that of the DKD and Chaga50 groups (12.7 ± 0.5 mg/g), but these differences were not statistically significant.

#### Effects of Chaga Extract on RBG

As shown in Figure 3C, at the baseline and 8th week of high-fat diet, the RBG among the four groups had no statistical difference (*p* = ns). After STZ injection, RBG levels at the 9th week in the DKD group (28.3 ± 0.6 mmol/L) and the two chaga groups (28.4 ± 0.8 mmol/L and 29.5 ± 0.5 mmol/L) were significantly higher than that of the control group (6.6 ± 0.1 mmol/L) (All: *p* < 0.001).

Compared with the DKD group, the significant decreasing effects of chaga on RBG could be seen in the Chaga50 group at the 13th and 17th week, and in the Chaga100 group at the 17th week (All: *p* < 0.001). There was no significant difference between the Chaga50 and Chaga100 groups at the 17th week (*p* = ns).

It could also be seen from Figure 3C that with the prolonged course of T2DM, the RBG at the 17th week was significantly higher than at the 9th week (*p* < 0.001). Conversely, the RBG at the 17th week in the Chaga100 group was significantly lower than at the 9th week (*p* = 0.009).

#### Effects of Chaga Extracts on Insulin Level and IRI

As shown in Figure 3D, the levels of fasting serum insulin of the DKD rats (24.2 ± 0.4 mIU/L) and the two chaga groups (24.0 ± 0.3 mIU/L and 24.6 ± 0.4 mIU/L) were significantly higher than that of the normal control (18.7 ± 0.2 mIU/L) after being fed with a different diet for 8 weeks (All: *p* < 0.01). At the 17th week, the blood insulin level of the DKD group was significantly lower than that of the control group (*p* = 0.020), but the insulin levels of Chaga50 and Chaga100 increased significantly compared with the DKD model (Both: *p* < 0.05), while there was no significant difference between the normal group and the Chaga100 group (*p* = ns).

The FBG was observed at the 8th and 17th week (data not shown). Then IRI was calculated using the HOMA-IR formula, and the results were shown in Figure 3E. At the 8th week, the IRIs of the DKD rats (5.7 ± 0.1) and the two chaga groups (5.8 ± 0.2 and 5.8 ± 0.1) were significantly higher than the IRI of the control group (4.5 ± 0.1) (Three: *p* < 0.05). It could also be seen from Figure 3E that with the prolonged course of T2DM, the IRI at the 17th week in the DKD group was significantly higher than that of the 8th week (*p* < 0.001).

Compared with the DKD rat group, the levels of IRI in the two chaga groups had no significant difference at the 8th week (*p* = ns). After 8 weeks intervention with chaga, the IRIs of the 50 and 100 mg/kg chaga groups (21.1 ± 0.8 and 21.5 ± 0.5) were significantly lower than that of the DKD model group (24.9 ± 0.7) (Both: *p* < 0.05).

#### Effects of Chaga Extracts on TG, TC and SCr Levels

At the 8th week, the levels of TG among the four groups had not shown any statistical difference after being fed with different diets for 8 weeks. At the end of the experiment, the level of TG in the DKD group still had not shown the statistical difference when compared with the normal control as well as the two chaga groups (data not shown).

As seen in Figure 3F, compared with the normal control group, the levels of TC in the DKD group and the two chaga groups were significantly increased after being fed with the high-fat diet for 8 weeks (*p* < 0.05, separately). Similar results were also presented at the 17th week (All: *p* < 0.001). At the 17th week, the TC level of the group treated with 100 mg/kg chaga was significantly lower than that of the DKD group as well as the group treated with 50 mg/kg group (*p* < 0.001). It could also be seen from Figure 3F that the TC level of the DKD group at the 17th week was significantly higher than at the 8th week (*p* < 0.001). Figure 3G showed the SCr results. At the 8th week, the SCr levels among the four animal groups had no statistical difference (*p* = ns). However, with the prolonged course of DKD, the SCr levels of the DKD model group displayed an increase from 61.3 ± 24.6 μmol/L at the 8th week to 97.0 ± 0.8 μmol/L at the 13th week (*p* ± 0.001), and further increased to 131.5 ± 2.6 μmol/L at the 17^th^ week (*p* ± 0.001).

At the 13th week, the SCr level of the DKD model group was significantly higher than that of the control group (*p* = 0.004). Compared to the DKD model, the two chaga groups did not show significant decreased effects on the SCr levels (Both: *p* = ns).

At the 17th week, the SCr level of the DKD model (131.5 ± 2.6 μmol/L) was still significantly higher than that of the control group (60.0 ± 4.3 μmol/L) (*p* < 0.001). The SCr level of the Chaga50 decreased to 82.1 ± 7.8 μmol/L and the Chaga100 decreased to 70.4 ± 5.3 μmol/L, and both showed significant differences compared to the DKD model (Both: *p* < 0.001). It is interesting to see the change in the SCr levels of the Chaga100 group during the experiment: SCr level increased significantly from the 8th week to the 13th week (*p* < 0.001), but decreased significantly from the 13th week to the 17th week (*p* < 0.001). Finally, the SCr level of the Chaga100 at the 17th week did not show any difference compared to that of the 8th week (*p* = ns). Furthermore, the SCr level of the Chaga100 had not shown the significant difference compared to that of the normal group at the 17th week (*p* = ns).

#### Effects of Chaga Extracts on Proteinuria

Figure 3H showed urinary protein excretion results. After eight weeks on different diets, 24 h urinary protein excretion among the four animal groups had no statistical difference. However, with the prolonged course of DKD, the urinary protein excretion of the DKD model group increased from 3.9 ± 0.4 mg/24 h at the 8th week to 20.7 ± 2.8 mg/24 h at the 13th week (*p* < 0.001), and further increased to 42.3 ± 2.2 mg/24 h at the 17th week (*p* < 0.001). At the 13th week, the urinary protein excretion of the DKD group (20.7 ± 2.8 mg/24 h) was significantly higher than that of the control group (5.4 ± 2.1 mg/24 h) (*p* < 0.001). Compared to the DKD model, the two chaga groups (19.5 ± 2.3 mg/24 h and 19.2 ± 2.0 mg/24 h) did not show significant decreased effects on the urinary protein excretion (Both: *p* = ns).

At the 17th week, the urinary protein excretion in the DKD group was still significantly higher than that of the control group (7.6 ± 1.0 mg/24 h, *p* < 0.001). After 8 weeks of intervention with chaga, the excretions in the two chaga groups (24.1 ± 2.2 mg/24 h and 34.1 ± 1.9 mg/24 h) were significantly lower than that of the DKD model group (Both: *p* < 0.001). The change in urinary protein in the Chaga100 increased from the 8th week to the 13th week (*p* < 0.001), but it did not change significantly from the 13th week to the 17th week (*p* = ns).

### Effects of Chaga Extracts on Renal Artery With Color Doppler Ultrasonography

**FIGURE 4:**
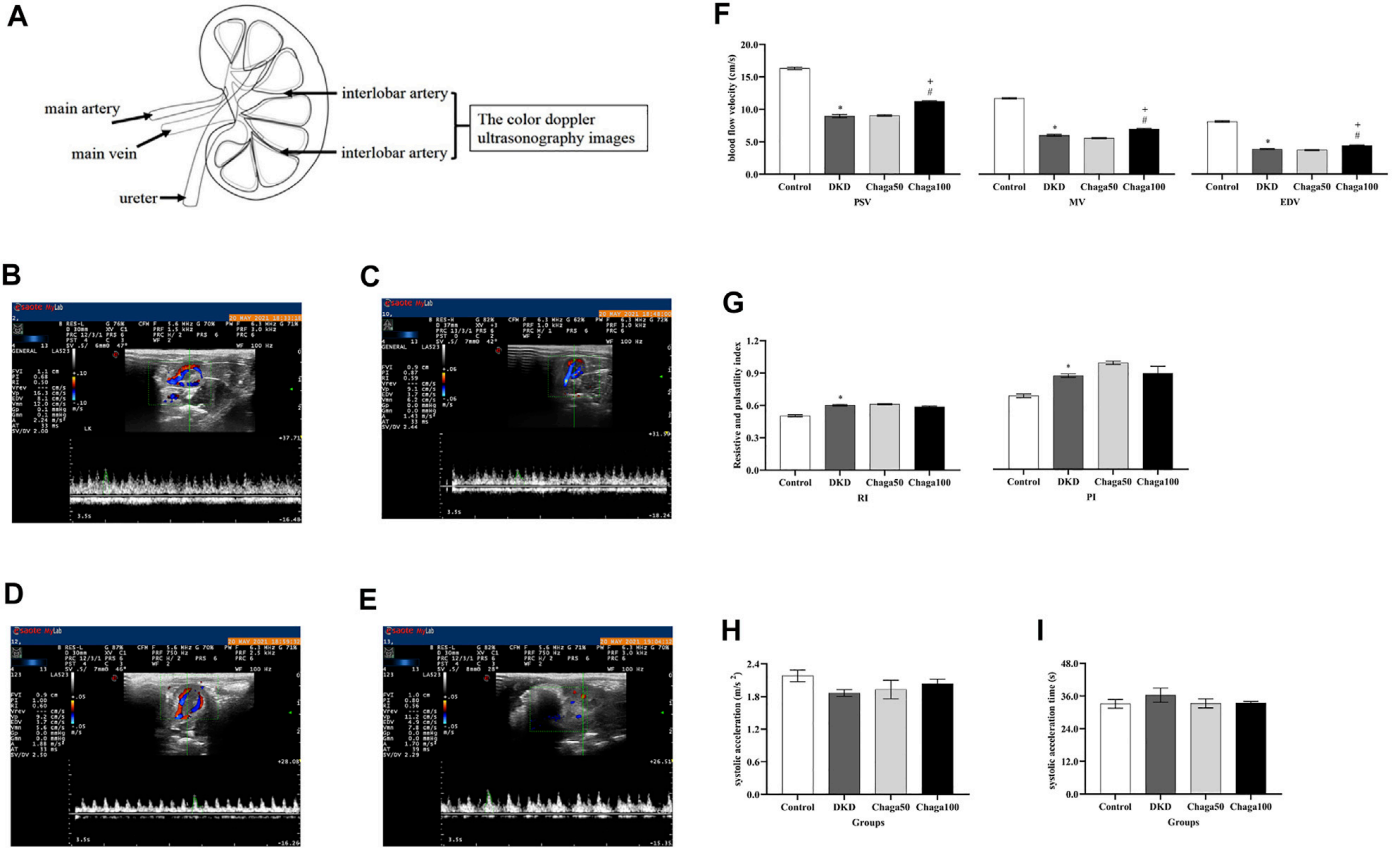
Anatomical location of the LRILA and the effects of chaga extracts on the LRILA with color Doppler ultrasonography. **(A)** The anatomical location of the renal interlobar artery. **(B–E)** Color Doppler ultrasonography images of LRILA of rats in each group. **(B)** Normal control group. **(C)** DKD model group. **(D)** Chaga50 group. **(E)** Chaga100 group. **(F–I)** Color Doppler ultrasonography parameters of LRILA of rats in each group. **(F)** peak systolic velocity (PSV), mean velocity (MV) and end-diastolic velocity (EDV) of the LRILA in different groups. **(G)** resistive index (RI) and pulsatility index (PI) of the LRILA in different groups. **(H)** systolic acceleration (SAC) of the LRILA in different groups. **(I)** systolic acceleration time (SAT) of the LRILA in different groups. Data were presented as mean ± SE. DKD, diabetic kidney disease; Chaga50, treatment with 50 mg/kg of chaga group; Chaga100, treatment with 100 mg/kg of chaga group; LRILA, left renal interlobar artery. *p < 0.05 vs. Normal control group, #*p* < 0.05 vs. DKD model group. + *p* < 0.05 vs. Chaga50 group.

Figure 4A showed the location of the left renal interlobar artery (LRILA). The color Doppler ultrasonography images of LRILA of rats in the normal control, DKD model, Chaga50, and Chaga100 were shown in Figures 4B–4E. Figure 4F showed that PSV, MV, and EDV of LRILA in the DKD group were significantly lower than those in the normal control (All: *p* < 0.001). The parameters above in the group treated with 100 mg/kg chaga were significantly higher than those in the DKD model group (All: *p* < 0.001) as well as the group treated with 50 mg/kg chaga (*p* < 0.001).

RI and PI in Figure 4G, SAC in Figure 4H, and SAT in Figure 4I of the DKD group, all showed significant differences compared to those of the normal control group(Four: *p* < 0.001). No significant effects could be seen in the two chaga groups on RI, PI, SAC, and SAT while they all were compared with the DKD rats (Eight: *p* = ns).

### Effects of Chaga Extracts on Renal Pathological Parameters

#### Histological Results With Light Microscopy

**FIGURE 5:**
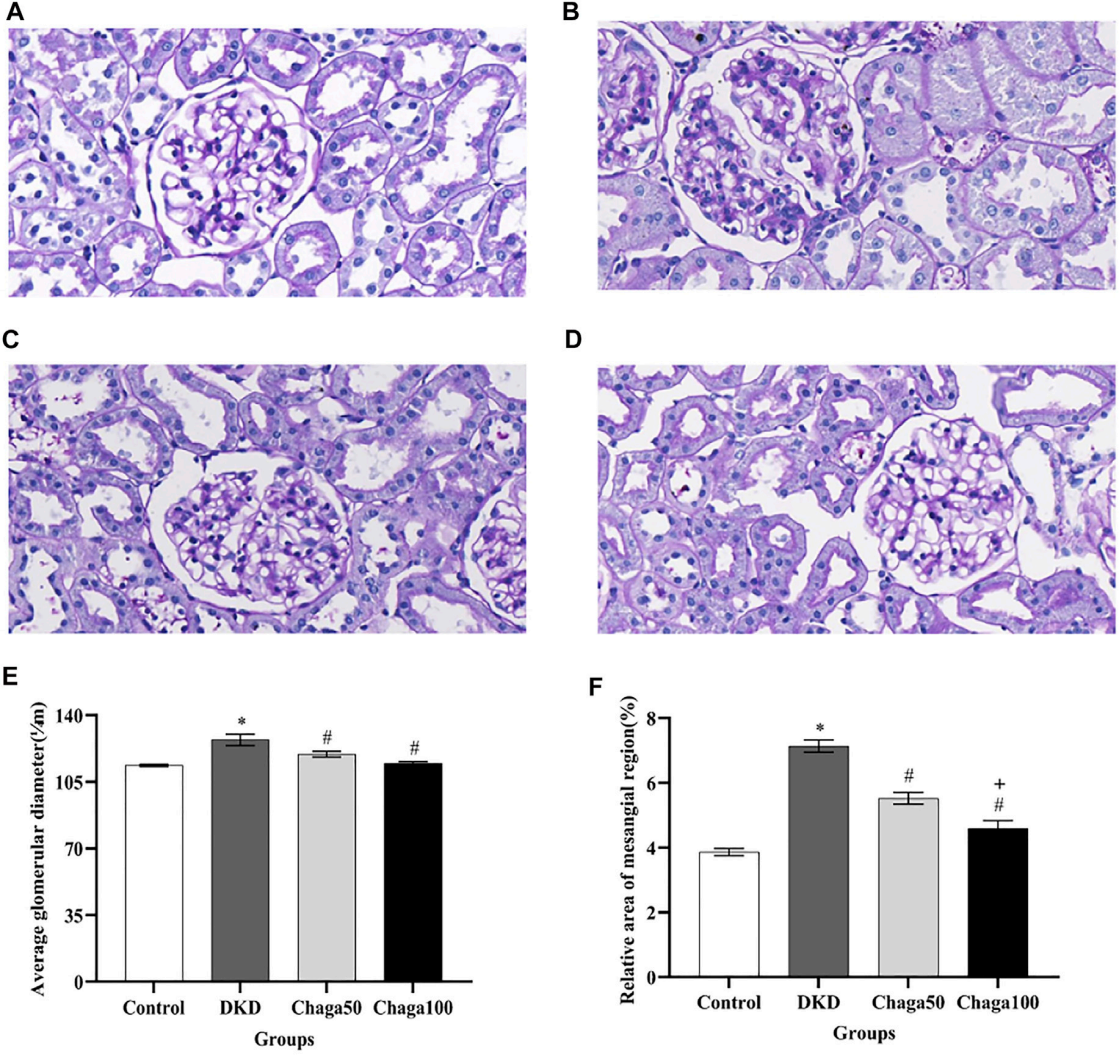
Effects of chaga on the pathological parameters of DKD rat model under the light microscope. **(A–D)** Light microscopic images of glomerulus (PAS staining × 400). **(A)** Normal control group. **(B)** DKD model group. **(C)** Chaga50 group. **(D)** Chaga100 group. **(E)** Average glomerular diameter of rats in each group. **(F)** Relative area of the mesangial region of rats in each group. Data were presented as mean ± SE. DKD, diabetic kidney disease; Chaga50, treatment with 50 mg/kg of chaga group; Chaga100, treatment with 100 mg/kg of chaga group. *p < 0.05 vs. Normal control group, #*p* < 0.05 vs. DKD group, + *p* < 0.05 vs. Chaga50 group.

Light microscopic images of the glomerulus (PAS staining × 400) were shown in Figure 5A for the normal control group, Figure 5B for the DKD model, Figure 5C for the Chaga50, and Figure 5D for the Chaga100 group. Histopathological PAS staining revealed that DKD rats exhibited obvious glomerular hypertrophy and mesangial matrix expansion compared to normal rats. Chaga markedly attenuated these renal pathological changes in a dose-dependent way.

The results in Figure 5E showed that the average glomerular size, which was represented as an average glomerular diameter, in the DKD group (127.1 ± 7.9 μm) was significantly larger than in the control group (113.6 ± 1.6 μm) (*p* < 0.001), while the sizes in the two intervention groups (119.5 ± 3.8 μm in the Chaga50 and 114.7 ± 2.6 μm in the Chaga100 group) were significantly smaller than in the DKD group (*p* < 0.05).

Figure 5F showed that compared with normal control group, the relative area of the mesangial region in the DKD model increased significantly (*p* < 0.05). The areas decreased in the two chaga groups significantly, compared to the DKD rats. And the results of the Chaga100 group were significantly smaller than that of the Chaga50 group (*p* = 0.01).

#### Histological Results With Electron Microscopy

**FIGURE 6:**
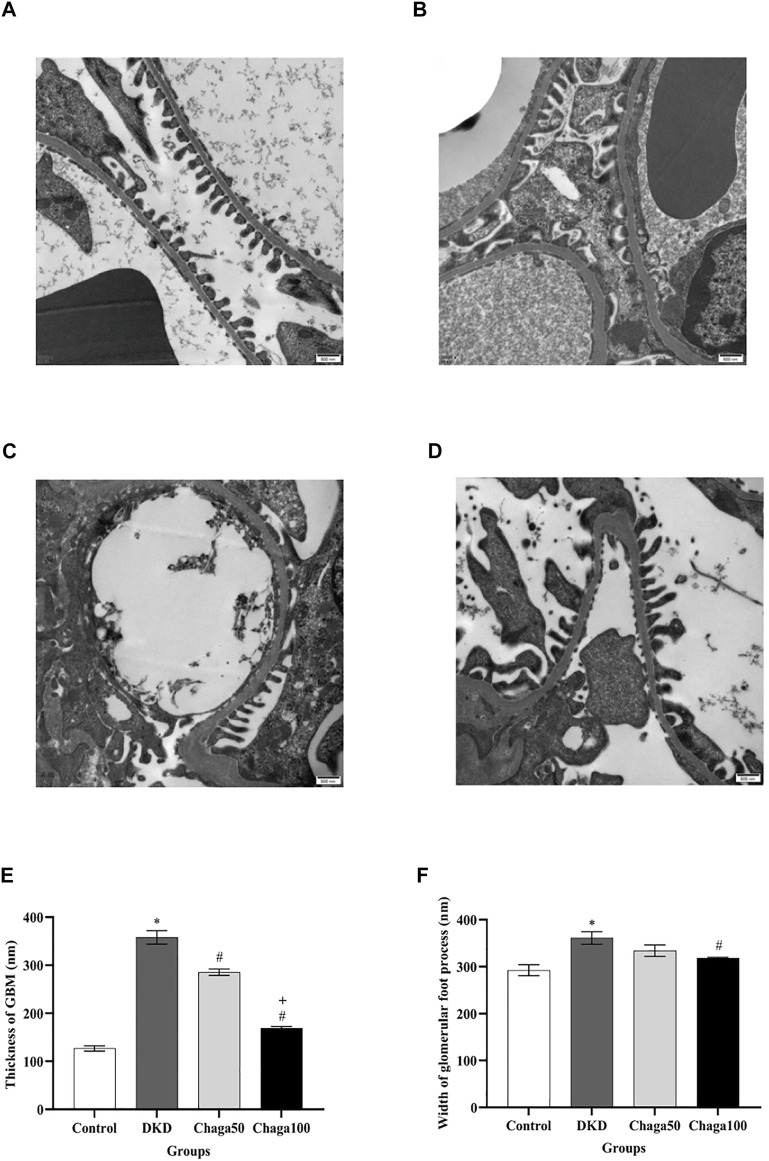
Effects of chaga on the pathological parameters of DKD rat model under the electron microscope. **(A–D)** Electron microscopic images of the basement membrane of the glomerulus and foot processes of podocytes (×20,000). **(A)** Normal control group. **(B)** DKD model group. **(C)** Chaga50 group. **(D)** Chaga100 group. **(E)** Thickness of GBM of rats in each group. **(F)** Foot process width of rats in each group. Data were presented as mean ± SE. DKD, diabetic kidney disease; Chaga50, treatment with 50 mg/kg of chaga group; Chaga100, treatment with 100 mg/kg of chaga group; GBM, glomerular basement membrane. *p < 0.05 vs. Normal control group, #*p* < 0.05 vs. DKD group, + *p* < 0.05 vs. Chaga50 group.

Figures 6A–6D showed electron microscopic images of the basement membrane of the glomerulus and foot processes of podocytes (×20,000) for the four groups are: normal control, DKD model, Chaga50, and Chaga100.

Figure 6E indicated that the average thickness of GBM in the DKD group was significantly larger than in the control group (*p* < 0.001), while the thickness in the two intervention groups was significantly smaller than in the DKD group (Both: *p* < 0.001), and the result of the Chaga100 was significantly smaller than that of the Chaga50 group (*p* < 0.001).

The foot processes of podocytes appeared to be segmental fusion and the average width of foot processes in the DKD group was larger than in the control group (*p* < 0.001). Compared with the DKD group, only the Chaga100 group showed a significant effect on decreasing the width of foot processes (*p* = 0.045). The results were shown in Figure 6F.

## Discussion

As a new type of medicinal fungus, chaga possesses antitumor, antioxidant, hypoglycemic, and hypolipidemic effects (Wang et al., 2017b; Yu et al., 2020; Duru et al., 2019; Burmasova et al., 2019; Lu et al., 2021). In recent years, the use of medicinal fungi in the treatment of kidney disease has been widely examined (Zhong et al., 2015; Wang et al., 2018). However, there are only a few reports about the influence of chaga on renal injury (Chou et al., 2016; Yong et al., 2018; Wang et al., 2017a). In this study, we established a DKD rat model induced by T2DM with the high-fat diet and low-dose STZ injection, and the treatment of chaga was observed.

The T2DM rat model was characterized by insulin resistance and hyperglycemia (Gheibi et al., 2017; Chao et al., 2018). During the progression of DM, the renal injury indexes including enhanced urinary protein and SCr were all observed in our study. According to the criteria of DKD diagnosis, glomerular hypertrophy, increased mesangial matrix, thickened GBM, and foot process fusion are the “Gold Standard” (Expert Group of Chinese Society of Nephrology, 2021). In order to determine the severity of kidney injury before the rats were sacrificed, we tried the RDUS technique, a noninvasive method, which plays an important role in the observation of the renal size, structure, and blood flow parameters clinically (Hoi et al., 2018; Petrucci et al., 2018). Our results demonstrated that 8 weeks of chaga administration showed remarkable hypoglycemic and hypolipidemic effects in high-fat diet/STZ-induced T2DM rats. Guided with biochemistry and LRILA, DM-induced renal injury was further assessed and the effects of chaga were observed. Finally, the results from light microscopy and electron microscopy confirmed the renal protection of chaga. Compared to the previous studies (Chou et al., 2016; Wang et al., 2017a), the present study showed more evidence based on biochemical parameters, RDUS, and electron microscopy.

The clinical features of DKD include an increase in persistent albumin excretion, and/or a progressive decrease in renal GFR, eventually leading to the development of ESRD (Expert Group of Chinese Society of Nephrology, 2021). DKD is a progressive disease, and the mortality of DKD increases with the progress of the disease (Sagoo and Gnudi, 2020). One of the current therapies is to slow down the progression of DKD (KDIGO Executive Committee, 2020; Expert Group of Chinese Society of Nephrology, 2021). SCr level is the most used biochemical parameter to estimate the progression of renal disease (Asmamaw et al., 2020). In our study, the evidence including SCr and urinary protein excretion indicated that kidney injury appeared at the 13th week and injury aggravation was shown at the 17th week based on SCr level. More findings were remarkable: the levels of RBG, SCr and urinary protein excretion did not show significant improvement after 4 weeks of continuous treatment with chaga, but the obvious effects were shown after another 4 weeks of being continuously treated with chaga. These results suggested that the protective effect of chaga might be related to delaying the deterioration of SCr, etc.

In addition, it has been reported that chaga extracts could reduce the blood lipid level, lipid accumulation, and body weight in DKD mice induced by the high-fat diet (Yu et al., 2020). This is consistent with the result of the present study that the body weights of the two intervention groups were lower than that of the DKD model group. However, the result is inconsistent with the two previous studies (Wang et al., 2017a; Yong et al., 2018), and the reasons were mainly due to the different drug components as a result of the different extraction methods of chaga were used, the different dose of drug intervention, and the different species of rodents were involved in testing.

As an effective, noninvasive, and repeatable method, Doppler ultrasound could rapidly and directly reveal the condition of renal hemodynamics, such as blood flow and vascular resistance (Abe et al., 2019). Deterioration of renal function can also be predicted with RDUS (Sener et al., 2018). According to the wave pattern of arterial blood flow in each region on renal Doppler ultrasonography, PSV, EDV, and MV of arterial flow can be measured (Abe et al., 2019). The levels of vascular resistance, also known as RI and PI, can be calculated based on PSV, EDV, and MV (Zou et al., 2017; Petrucci et al., 2018).

The impairment of PSV, MV, EDV, PI, and RI of the renal artery would affect renal physiology and functions. The progression of chronic kidney disease was accompanied by a decline in the renal artery blood flow velocity and an increase in renal artery resistance (Yang et al., 2021). It is suggested that EDV and RI may be the best two indexes to evaluate renal function (Abe et al., 2019; Di Nicolo and Granata, 2019). It has been reported that the PSV and EDV of the intrarenal artery were significantly positively correlated with renal function (Gao et al., 2017), while RI and PI were negatively correlated with renal function (Sener et al., 2018). In addition, the variation of the MV is more sensitive which evolves with the hemodynamic resistance following the Poiseuille law (Zheng and Qin, 2017). With the Doppler ultrasound technique, our research suggested that renal function was impaired by the significantly decreased PSV, MV, EDV and increased PI and RI in the DKD group. On the other hand, the protection of chaga was also confirmed from the points of significantly increased PSV, MV, and EDV.

The above evidence from RDUS indicated significant renal injury before the pathological confirmations. Our results suggested that, as a certain predictive indicating index in clinical practice (Meyer et al., 2021), RDUS showed its advantage in evaluating renal injury in the DKD rat model. Our RDUS results also suggested that the improvement of interlobar artery PSV, MV, and EDV were consistent with the improvement of renal pathology in DKD rats after chaga intervention.

The study also found that the average thickness of GBM, segmental fusion of podocytes and the average width of foot processes were all improved after chaga intervention. Electron microscopy is a mainstay in the analysis of renal biopsies, where it is typically employed in a correlative fashion along with light and immunofluorescence microscopy (Haas et al., 2020; Howell and Herrera, 2021). Electron microscopy can identify the lesions of glomerular podocytes better than light microscopy, which is helpful for further study about podocyte injury of DKD (Haas et al., 2020). The present study showed first the renal protective effect of chaga based on electron microscopy.

Based on biochemical indicators such as RBG, IRI, TC, SCr and urinary protein excretion above, color Doppler ultrasound assessment on PSV, EDV, MV, RI, PI and histopathological evidence including PAS light and electron microscopy, the renal protective effects of chaga on T2DM-induced renal injury were confirmed.

Total phenols, flavonoids and polysaccharides were analyzed in this study. Till now, the renal protective effects of chaga are mostly attributed to its polysaccharide (Chou et al., 2016; Wang et al., 2017a). Our research confirmed that the content of total polysaccharide was the highest among the three ingredients investigated. This is consistent with the results of some previous studies (Duru et al., 2019; Lu et al., 2021). Like polysaccharides, both phenols and flavonoids obtained from other mushrooms already showed their effects on diabetes and DKD (Onuekwuzu et al., 2019; Wang et al., 2019). The antidiabetic effect of some flavonoids from *Pleurotus tuberregium* was observed in alloxan-induced diabetic rabbits (Onuekwuzu et al., 2019). The renoprotective activities of phenolic meroterpenoids from *Ganoderma cochlear* were evaluated using rat renal interstitial fibroblast cells (Wang et al., 2019). Additionally, phenolic compounds and flavonoids from chaga also played important roles in antioxidant and hypoglycemic functions (Duru et al., 2019; Poyedinok et al., 2020). Further research of purified polysaccharides, active phenols or flavonoids from chaga should be conducted on renal protection separately. These results might be very interesting and important to the future use of chaga in DKD. Moreover, further chemical experiments are required in order to elucidate the chemical structure of the active compounds in the extract.

The limitation of the study is that the color Doppler ultrasound was not performed while SCr and proteinuria results already showed renal injury at the 13th week. If the images at the 13th and the 17th week could be compared, it would help us to understand the aggravation of renal injury from renal blood flow velocity and the influence of chaga.

Moreover, the level of serum α-klotho decreased in the DKD group, meanwhile, it increased in the chaga-treated groups in this study (data not shown). It was recently discovered that α-klotho can alleviate podocyte injury by inhibiting calcium ions influx mediated by transient receptor potential channel 6 (Kim et al., 2017). Research on the pathway α-klotho involved is being carried out, and related results may be presented in our follow-up articles.

In conclusion, our experiments conducted on the DKD rat model indicated that RDUS is an effective, noninvasive, and repeatable method to evaluate renal injury. The renal protective effects of chaga are confirmed by biochemical, color Doppler ultrasound, and histopathological evidence. Additionally, the protection of chaga is related to its effects on delaying the progression of renal injury. Furthermore, our research might provide a valuable basis for the clinical application of chaga in the prevention and treatment of DKD.

## Data Availability

The original contributions presented in the study are included in the article/Supplementary Material, further inquiries can be directed to the corresponding author.

## References

[B1] Abe M. Akaishi T. Miki T. Miki M. Funamizu Y. Araya K. (2019). Influence of Renal Function and Demographic Data on Intrarenal Doppler Ultrasonography. PLoS One 14 (8), e0221244. 10.1371/journal.pone.0221244 31454365 PMC6711528

[B2] Asmamaw T. Genet S. Menon M. Tarekegn G. Chekol E. Geto Z. (2020). Early Detection of Renal Impairment Among Patients with Type 2 Diabetes Mellitus through Evaluation of Serum Cystatin C in Comparison with Serum Creatinine Levels: a Cross-Sectional Study. Diabetes Metab. Syndr. Obes. 13, 4727–4735. 10.2147/DMSO.S279949 33299336 PMC7721116

[B3] Azushima K. Gurley S. B. Coffman T. M. (2018). Modelling Diabetic Nephropathy in Mice. Nat. Rev. Nephrol. 14 (1), 48–56. 10.1038/nrneph.2017.142 29062142

[B4] Brosius F. C. Alpers C. E. Bottinger E. P. Breyer M. D. Coffman T. M. Gurley S. B. (2009). Mouse Models of Diabetic Nephropathy. J. Am. Soc. Nephrol. 20 (12), 2503–2512. 10.1681/ASN.2009070721 19729434 PMC4075053

[B5] Burmasova M. A. Utebaeva A. A. Sysoeva E. V. Sysoeva M. A. (2019). Melanins of Inonotus Obliquus: Bifidogenic and Antioxidant Properties. Biomolecules 9 (6), 248. 10.3390/biom9060248 PMC6628194 31238558

[B6] Chao P. C. Li Y. Chang C. H. Shieh J. P. Cheng J. T. Cheng K. C. (2018). Investigation of Insulin Resistance in the Popularly Used Four Rat Models of Type-2 Diabetes. Biomed. Pharmacother. 101, 155–161. 10.1016/j.biopha.2018.02.084 29486333

[B7] Chou Y. J. Kan W. C. Chang C. M. Peng Y. J. Wang H. Y. Yu W. C. (2016). Renal Protective Effects of Low Molecular Weight of Inonotus Obliquus Polysaccharide (LIOP) on HFD/STZ-induced Nephropathy in Mice. Int. J. Mol. Sci. 17 (9), 1535. 10.3390/ijms17091535 PMC5037810 27649140

[B8] Di Nicolò P. Granata A. (2019). Renal Intraparenchymal Resistive index: the Ultrasonographic Answer to many Clinical Questions. J. Nephrol. 32 (4), 527–538. 10.1007/s40620-018-00567-x 30539416

[B9] Duru K. C. Kovaleva E. G. Danilova I. G. van der Bijl P. (2019). The Pharmacological Potential and Possible Molecular Mechanisms of Action of Inonotus Obliquus from Preclinical Studies. Phytother. Res. 33 (8), 1966–1980. 10.1002/ptr.6384 31209936

[B10] Expert Group of Chinese Society of Nephrology (2021). Chinese Guidelines for Diagnosis and Treatment of Diabetic Kidney Disease. Chin. J. Nephrol. 37 (3), 255–304. 10.3760/cma.j.cn441217-20201125-00041

[B11] Fanali C. Gallo V. Della Posta S. Dugo L. Mazzeo L. Cocchi M. (2021). Choline Chloride-Lactic Acid-Based NADES as an Extraction Medium in a Response Surface Methodology-Optimized Method for the Extraction of Phenolic Compounds from Hazelnut Skin. Molecules 26 (9), 2652. 10.3390/molecules26092652 34062718 PMC8125409

[B12] Gao J. Perlman A. Kalache S. Berman N. Seshan S. Salvatore S. (2017). Multiparametric Quantitative Ultrasound Imaging in Assessment of Chronic Kidney Disease. J. Ultrasound Med. 36 (11), 2245–2256. 10.1002/jum.14209 28407281 PMC5640470

[B13] Gheibi S. Kashfi K. Ghasemi A. (2017). A Practical Guide for Induction of Type-2 Diabetes in Rat: Incorporating a High-Fat Diet and Streptozotocin. Biomed. Pharmacother. 95, 605–613. 10.1016/j.biopha.2017.08.098 28881291

[B14] Giralt-López A. Molina-Van den Bosch M. Vergara A. García-Carro C. Seron D. Jacobs-Cachá C. (2020). Revisiting Experimental Models of Diabetic Nephropathy. Int. J. Mol. Sci. 21 (10), 3587. 10.3390/ijms21103587 PMC7278948 32438732

[B15] Haas M. Seshan S. V. Barisoni L. Amann K. Bajema I. M. Becker J. U. (2020). Consensus Definitions for Glomerular Lesions by Light and Electron Microscopy: Recommendations from a Working Group of the Renal Pathology Society. Kidney Int. 98 (5), 1120–1134. 10.1016/j.kint.2020.08.006 32866505

[B16] Hoi S. Takata T. Sugihara T. Ida A. Ogawa M. Mae Y. (2018). Predictive Value of Cortical Thickness Measured by Ultrasonography for Renal Impairment: a Longitudinal Study in Chronic Kidney Disease. J. Clin. Med. 7 (12). 10.3390/jcm7120527 PMC6306756 30544567

[B17] Howell D. N. Herrera G. A. (2021). Electron Microscopy in Renal Pathology: Overall Applications and Guidelines for Tissue, Collection, Preparation, and Stains. Ultrastruct. Pathol. 45 (1), 1–18. 10.1080/01913123.2020.1854407 33320036

[B18] Huang Y. Xu J. Wu X. Chen X. Bai X. Zhuang Y. (2019). High Expression of Complement Components in the Kidneys of Type 2 Diabetic Rats with Diabetic Nephropathy. Front. Endocrinol. (Lausanne) 10, 459. 10.3389/fendo.2019.00459 31338070 PMC6629834

[B19] KDIGO Executive Committee (2020). KDIGO 2020 Clinical Practice Guideline for Diabetes Management in Chronic Kidney Disease. Kidney Int. 98 (4S), S1–S115. 10.1016/j.kint.2020.06.019 32998798

[B20] Kim J. H. Xie J. Hwang K. H. Wu Y. L. Oliver N. Eom M. (2017). Klotho May Ameliorate Proteinuria by Targeting TRPC6 Channels in Podocytes. J. Am. Soc. Nephrol. 28 (1), 140–151. 10.1681/ASN.2015080888 27151926 PMC5198269

[B21] Li N. Wang Y. R. Tian X. Q. Lin L. Liang S. Y. Li Q. Y. (2020). Potential Value of Three-Dimensional Ultrasonography in Diagnosis of Diabetic Nephropathy in Chinese Diabetic Population with Kidney Injury. BMC Nephrol. 21 (1), 243. 10.1186/s12882-020-01902-w 32600283 PMC7325142

[B22] Liao H. Jia D. Zhao X. Zheng D. Li Y. Li R. (2020). Effects of Chaga Medicinal Mushroom *Inonotus Obliquus* (Agaricomycetes) Extracts on NOS-cGMP-PDE5 Pathway in Rat Penile Smooth Muscle Cells. Int. J. Med. Mushrooms. 22 (10), 979–990. 10.1615/IntJMedMushrooms.2020035812 33426827

[B23] Lu Y. Jia Y. Xue Z. Li N. Liu J. Chen H. (2021). Recent Developments in Inonotus Obliquus (Chaga Mushroom) Polysaccharides: Isolation, Structural Characteristics, Biological Activities and Application. Polymers 13 (9), 1441. 10.3390/polym13091441 33947037 PMC8124789

[B24] Meyer S. Fuchs D. Meier M. (2021). Ultrasound and Photoacoustic Imaging of the Kidney: Basic Concepts and Protocols. Methods Mol. Biol. 2216, 109–130. 10.1007/978-1-0716-0978-1_7 33475997 PMC9703212

[B25] Naidoo P. Islam M. S. (2014). Development of an Alternative Non-obese Non-genetic Rat Model of Type 2 Diabetes Using Caffeine and Streptozotocin. Pharmacol. Rep. 66 (4), 585–593. 10.1016/j.pharep.2014.02.019 24948058

[B26] Onuekwuzu I. M. Chidinma I. C. Chigozie I. J. (2019). Anti-diabetic Effect of a Flavonoid and Sitosterol - Rich Aqueous Extract of Pleurotus Tuberregium Sclerotia in Alloxan-Induced Diabetic Rabbits. Endocr. Metab. Immune Disord. Drug Targets 19 (8), 1148–1156. 10.2174/1871530319666190206213843 30727935

[B27] Petrucci I. Clementi A. Sessa C. Torrisi I. Meola M. (2018). Ultrasound and Color Doppler Applications in Chronic Kidney Disease. J. Nephrol. 31 (6), 863–879. 10.1007/s40620-018-0531-1 30191413

[B28] Poyedinok N. Mykhaylova O. Sergiichuk N. Tugay T. Tugay A. Lopatko S. (2020). Effect of Colloidal Metal Nanoparticles on Biomass, Polysaccharides, Flavonoids, and Melanin Accumulation in Medicinal Mushroom Inonotus Obliquus (Ach.:Pers.) Pilát. Appl. Biochem. Biotechnol. 191 (3), 1315–1325. 10.1007/s12010-020-03281-2 32096061

[B29] Sagoo M. K. Gnudi L. (20202067). Diabetic Nephropathy: an Overview. Methods Mol. Biol. 2067, 3–7. 10.1007/978-1-4939-9841-8_1 31701441

[B30] Sener T. E. Tanidir Y. Bin Hamri S. Sever I. H. Ozdemir B. Al-Humam A. (2018). Effects of Flexible Ureteroscopy on Renal Blood Flow: a Prospective Evaluation. Scand. J. Urol. 52 (3), 213–218. 10.1080/21681805.2018.1437770 29463207

[B31] Song H. T. Gao Y. Yang Y. M. Xiao W. J. Liu S. H. Xia W. C. (2016). Synergistic Effect of Cellulase and Xylanase during Hydrolysis of Natural Lignocellulosic Substrates. Bioresour. Technol. 219, 710–715. 10.1016/j.biortech.2016.08.035 27560367

[B32] Wang C. Hou X.-x. Rui H.-l. Li L.-j. Zhao J. Yang M. (20182018). Artificially Cultivated Ophiocordyceps Sinensis Alleviates Diabetic Nephropathy and its Podocyte Injury via Inhibiting P2X7R Expression and NLRP3 Inflammasome Activation. J. Diabetes Res. 2018, 1–16. 10.1155/2018/1390418 PMC6252193 30534570

[B33] Wang J. Hu W. Li L. Huang X. Liu Y. Wang D. (2017a). Antidiabetic Activities of Polysaccharides Separated from Inonotus Obliquus via the Modulation of Oxidative Stress in Mice with Streptozotocin-Induced Diabetes. PLoS One 12 (6), e0180476. 10.1371/journal.pone.0180476 28662169 PMC5491251

[B34] Wang J. Wang C. Li S. Li W. Yuan G. Pan Y. (2017b). Anti-diabetic Effects of Inonotus Obliquus Polysaccharides in Streptozotocin-Induced Type 2 Diabetic Mice and Potential Mechanism via PI3K-Akt Signal Pathway. Biomed. Pharmacother. 95, 1669–1677. 10.1016/j.biopha.2017.09.104 28954386

[B35] Wang P. C. Zhao S. Yang B. Y. Wang Q. H. Kuang H. X. (2016). Anti-diabetic Polysaccharides from Natural Sources: A Review. Carbohydr. Polym. 148, 86–97. 10.1016/j.carbpol.2016.02.060 27185119

[B36] Wang X. L. Wu Z. H. Di L. Zhou F. J. Yan Y. M. Cheng Y. X. (2019). Renoprotective Phenolic Meroterpenoids from the Mushroom Ganoderma Cochlear. Phytochemistry 162, 199–206. 10.1016/j.phytochem.2019.03.019 30947089

[B37] Yang J. Yang S. Xu Y. Lu F. You L. He Z. (2021). Evaluation of Renal Oxygenation and Hemodynamics in Patients with Chronic Kidney Disease by Blood Oxygenation Level-dependent Magnetic Resonance Imaging and Intrarenal Doppler Ultrasonography. Nephron 145 (6), 653–663. 10.1159/000516637 34182563

[B38] Yong T. Chen S. Liang D. Zuo D. Diao X. Deng C. (2018). Actions of Inonotus Obliquus against Hyperuricemia through XOD and Bioactives Screened by Molecular Modeling. Int. J. Mol. Sci. 19 (10). 10.3390/ijms19103222 PMC6214139 30340390

[B39] Yu J. Xiang J. Y. Xiang H. Xie Q. (2020). Cecal Butyrate (Not Propionate) Was Connected with Metabolism-Related Chemicals of Mice, Based on the Different Effects of the Two Inonotus Obliquus Extracts on Obesity and Their Mechanisms. ACS Omega 5 (27), 16690–16700. 10.1021/acsomega.0c01566 32685836 PMC7364710

[B40] Zhao L. Ji Z. Li K. Wang B. Zeng Y. Tian S. (2020). HPLC-DAD Analysis of Hyssopus Cuspidatus Boriss Extract and Mensuration of its Antioxygenation Property. BMC Complement. Med. Ther. 20 (1), 228. 10.1186/s12906-020-03016-0 32689984 PMC7370466

[B41] Zheng H., P. Qin R. J. (2017). Comparative Analysis of Hemorheology Property of Bloodin Vitroandin Vivo. Chin. J. Med. Phys. 34 (10), 1051–1057. 10.3969/j.issn.1005-202X.2017.10.017

[B42] Zhong D. Wang H. Liu M. Li X. Huang M. Zhou H. (2015). Ganoderma Lucidum Polysaccharide Peptide Prevents Renal Ischemia Reperfusion Injury via Counteracting Oxidative Stress. Sci. Rep. 5, 16910. 10.1038/srep16910 26603550 PMC4658483

[B43] Zou C. Jiao Y. Li X. Wang P. Zheng J. Zhao Y. (2017). Differences between Healthy Adults and Patients with Type 2 Diabetes Mellitus in Reactivity of Toe Microcirculation by Ultrasound Combined with a Warm bath Test. Medicine (Baltimore) 96 (22), e7035. 10.1097/MD.0000000000007035 28562559 PMC5459724

